# Comparative Study of Three Carbon Additives: Carbon Nanotubes, Graphene, and Fullerene-C60, for Synthesizing Enhanced Polymer Nanocomposites

**DOI:** 10.3390/nano10050838

**Published:** 2020-04-27

**Authors:** Xingyu Wang, Fujian Tang, Qi Cao, Xiaoning Qi, Matthew Pearson, Mingli Li, Hong Pan, Zi Zhang, Zhibin Lin

**Affiliations:** 1 Department of Civil and Environmental Engineering, North Dakota State University, Fargo, ND 58018, USA; xingyu.wang@ndsu.edu (X.W.); matthew.w.pearson@ndsu.edu (M.P.); mingli.li@ndsu.edu (M.L.); hong.pan@ndsu.edu (H.P.); zi.zhang@ndsu.edu (Z.Z.); 2 State Key Laboratory of Coastal and Offshore Engineering, School of Civil Engineering, Dalian University of Technology, Dalian 116024, China; ftang@dlut.edu.cn (F.T.); qcao@dlut.edu.cn (Q.C.); 3 Department of Coatings and Polymeric Materials, North Dakota State University, Fargo, ND 58018, USA; xiaoning.qi@ndsu.edu

**Keywords:** nano-modified coating, nanocomposite, graphene nanoplatelets, carbon nanotube, fullerene-C60, corrosion resistance

## Abstract

While nanoparticles from the carbon family have been incorporated effectively for polymer matrixes, there is no clear information available for understanding the impacts of the morphology of different carbon nanoparticles on the performance of carbon-based nanocomposites. Therefore, this study aimed to provide a comprehensive, comparative investigation to systematically assess the impacts of nanoparticles on the tribological, mechanical, and electrochemical properties of the epoxy coatings using three representative 0D, 1D, and 2D nanoparticles: Fullerene-C60 (C60), graphene nanoplatelets (GNPs), and carbon nanotubes (CNTs). The anti-corrosion performance of the nanocomposites in both the short and long term was characterized. The mechanical properties were examined by abrasion, adhesion, and tensile tests. Fourier-transform infrared spectroscopy (FTIR) was conducted to determine their chemical structures, while scanning electron microscopy (SEM) was used to determine their surface texture. The electrochemical impedance spectroscopy (EIS) results revealed that the coatings reinforced by C60 and GNP had better anti-corrosion performance than that of the CNT/epoxy samples. The incorporation of C60 and CNT led to a considerable improvement in tensile properties, while improved abrasion resistance was observed in all types of nanofiller/epoxy groups. C60-loaded composites exhibited a significant enhancement in tensile properties as compared to CNT or GNP composites.

## 1. Introduction

Nanomaterials, due to their excellent physical, electrical, and other proprieties, are widely accepted as a building block for broader applications [1]. Among them, the carbon family, carbon-based nanoparticles, exhibits unique characteristics to serve as nanofillers for synthesizing multifunctional nanocomposites with dramatically enhanced performance [2]. Particularly, a wide range of carbon families covering different morphologies and sizes provides broad selections for different fields of interest [3,4]. Currently, carbon-based nanoparticles are mainly classified as zero-dimensional (0D), one-dimensional (1D), or two-dimensional (2D) nanomaterials [5]. Three typical nanoparticles, fullerene-C60 (C60), carbon nanotubes (CNTs), and graphene nanoplatelets (GNPs) [6,7], were selected in this study, and they represent 0D,1D, and 2D materials, respectively. Graphene (GNP) is a crystalline structure and two-dimensional sheet structure, only consisting of in-plane hexagonal rings of carbons. By contrast, carbon nanotubes (CNT) exhibit cylindrical structures, similar to a rolled graphene sheet with a nanoscale diameter [8]. Fullerene-C60 is a 0D cage-like spherical structure with 20 hexagons and 12 pentagons and is also used as a nanofiller [9].

The inclusion of CNTs, GNPs, or C60 for synthesizing nanocomposite coatings has recently received wide attention [10,11,12,13,14,15,16,17,18,19,20,21,22,23]. Their unique shapes assisted polymeric composites in improving their performance [16]. Researchers reported that the incorporation of CNTs resulted in enhanced mechanical properties, while the nanocomposite with GNPs exhibited improved corrosion resistance. Fullerene-C60 nanocomposites exhibited reduced friction, toughened mechanical properties, and improved anti-corrosion behavior, as compared to the neat polymer materials [9,17,18]. Research on the CNT/polymer composites focused on their mechanical properties and electrical conductivity. Jeon et al. [10] assembled a CNT/epoxy coating with nanofiller contents of 0, 0.25, and 0.5 wt.%. Their results showed that there was an increase in adhesion strength of the coating and a decrease in the impedance modulus for corrosion potential. Cui et al. [12] discussed the impacts of functionalized CNTs in the tensile strength of epoxy coatings, and the tensile strength increased by 57% compared to neat epoxy. Unlike previous work, Zabet et al. [14] found that the addition of CNT caused a reduction in mechanical properties due to the curing process. Their results indicated that the hardness and elastic modulus were significantly reduced in the samples with 2.8 wt.% of CNT. By contrast, researchers mainly studied the anti-corrosion performance of graphene/polymer coatings [19,20,21,22,23]. Liu et al. [19] investigated graphene-reinforced epoxy coatings with 0.5 and 1.0 wt.% of GNP nanosheets. Improved anti-corrosion performance was observed in the GNP/epoxy coating and stronger reinforcement was obtained in the specimens containing 0.5 wt.% of GNPs. Other researchers [22,23,24] found that a higher amount of GNP nanofillers could still effectively enhance the anti-corrosion and barrier properties of coating films. Monetta et al. [23] fabricated graphene/epoxy nanocomposites and observed that the addition of 1.0 wt.% of GNP nanoparticles could provide a strong improvement in corrosion resistance performance.

Meanwhile, fullerene-C60 (C60) nanoparticles served as nanofillers due to the unique spherical structure with a nanosized diameter to reinforce the polymer matrix [25,26]. Compared to CNTs and GNPs, the presence of C60 nanoparticles improved both corrosion resistance and mechanical properties [9]. Liu et al. [9] investigated the fullerene-C60-reinforced epoxy with varied content of C60 from 0.25 to 1.0 wt.%, and the C60/epoxy nanocomposites showed improved corrosion resistance and tribological properties. In addition, 0.5 wt.% of C60 particles provided the strongest reinforcement; thus, degradation was observed in the nanocomposites with a higher content of C60, which was correlated with the increased degree of aggregation of nanoparticles. Pikhurow et al. [17] evaluated the mechanical properties of C60-reinforced nanocomposites, and they concluded that 0.08 wt.% of fullerene-C60 additions achieved a great improvement in both tensile strength and Young’s modulus. Obviously, many investigations focused on a single specific property to address nanofiller enhancement, but very few of the published literature provided a comprehensive, systematic assessment to quantify the mechanical and electrochemical properties associated with nanoparticles. The findings from those documents could not provide a competitive suggestion for the selection or application of nanoparticles, due to different contents and dispersion methods used from different researchers [1,2,3,4,5,6,7,8,9,10,11,12,13,14,15,16,17,18,19,20,21].

To the best of the authors knowledge, there is still a lack of research to comparatively evaluate the influence of geometric shape for the carbon-based nanofillers on nano-reinforcement, especially an experimental investigation of the tribological, mechanical, and electrochemical properties. Three representative carbon nanoparticles, CNT, GNP, and C60, were selected as additives for reinforcing the epoxy coating, and the major focus was on their enhancements on the tribological, mechanical, and electrochemical properties. The nanocomposites were fabricated using an identical mixture design and an identical dispersion procedure to generate a comparative situation to better determine the effects of different nanoparticles on their performance. The influence of the inclusion of the nanofillers in the polymeric coating was investigated in detail by: (i) Potentiostatic EIS test, (ii) Taber abraser test, and (iii) coupon tensile test. SEM and transmission electron microscopy (TEM) were used to characterize micro-scale information of the nanofillers and the nanocomposites.

## 2. Materials and Methods

This section describes the detailed experimental investigation for providing a comprehensive comparative study of incorporating three different nanomaterials into the coating. Detailed information of the materials and characterization methods are presented in detail below.

### 2.1. Materials

**Figure 1:**
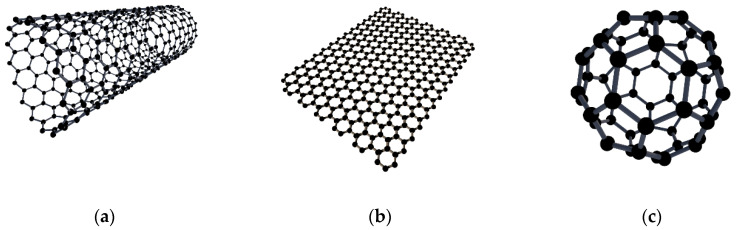
Atom structure of (**a**) carbon nanotubes, (**b**) graphene nanoplatelets, and (**c**) fullerene-C60 particles.

As stated, three different carbon-based nanofillers were selected, and the atom structures of CNT, GNP, and fullerene-C60 are illustrated in Figure 1. In this study, all chemicals were purchased commercially and used as received without any modification. EPON™ Resin 828 was used as a bisphenol A epoxy resin and the curing agent was Epikure 3175, which were both obtained from Hexion Inc. (Columbus, OH, USA). Carbon nanotubes, graphene nanoplatelets, and fullerene-C60 were used as nanofillers to reinforce the polymer matrix. The length of the multi-walled carbon nanotubes (Cheap Tubes Inc., Cambridgeport, VT, USA) was around 10–50 µm, with an outer diameter of 8–15 nm. The graphene nanoplatelets had an average thickness of 8–12 nm (Cheap Tubes Inc., Cambridgeport, VT, USA), and the fullerene-C60 particles (Sigma-Aldrich Corp., St. Louis, MO, USA) had an average diameter around 20 nm.

### 2.2. Fabrication of Nanocarbon-Reinforced Epoxy Composites

**Figure 2:**
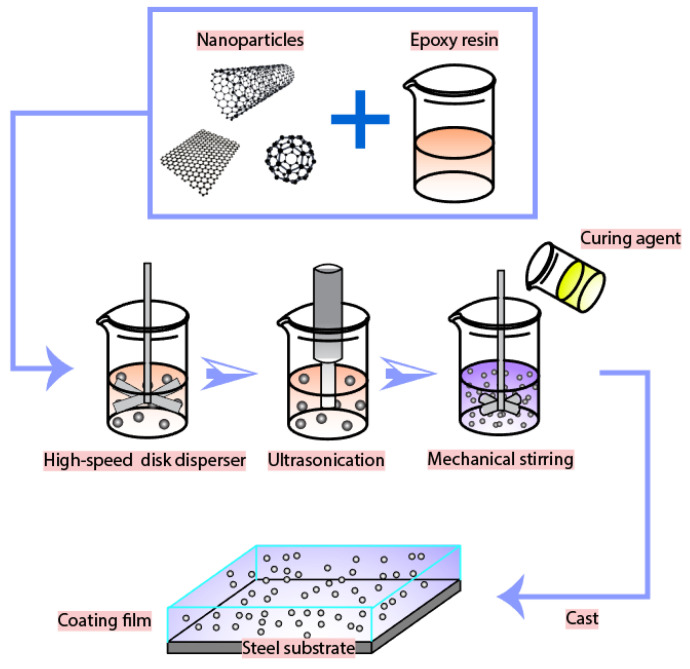
Schematic of the fabrication process.

The fabrication procedure of coating systems is illustrated in Figure 2, where the nanofillers were dispersed into epoxy resin by utilizing a high-speed disk (HSD) disperser and ultra-sonication. As presented in Figure 2, nanoparticles were mixed in epoxy resin and subjected to high-speed disk dispersion for 30 min, and the rotation speed was fixed at 4000 rpm. To improve the dispersion level, a 3/4” probe was used to ultra-sonicate the obtained mixture for 60 min, with the application of a 30 s on/off cycle. During the HSD and ultrasonication dispersion, the mixture was kept in a water/ice bath to avoid overheating. Subsequently, Epikure 3175 curing agent was added to the slurry while mechanical stirring was applied, the duration of mixing was 10 min, and the rotational speed was 600 rpm; thus, the mole ratio between EPON 828 and Epikure 3175 was 1:1. Finally, the coatings were applied on substrates and then left to be cured for 24 h at room temperature. Weight ratios of 0.1, 0.5, 1.0, 1.5, and 3.0 wt.% of the nanofillers were added to the nanocomposites to fabricate test specimens. All the nanocomposite coatings were denoted regarding the type and weight concentration of nanofiller. For example, 0.1%CNT-epoxy, 0.1%GNP-epoxy, and 0.1%C60-epoxy labeled the composites containing 0.1 wt.% of carbon nanotubes, graphene, and fullerene-C60, respectively. Simultaneously, the neat epoxy specimens were utilized as the baseline.

### 2.3. Characterization of the New Composite Coatings

#### 2.3.1. Corrosion Resistance

The corrosion barrier performance of the coatings was evaluated by EIS test. The coating films were cast on S-36 steel panels (Q-Lab Corp., Cleveland, OH, USA) and a three-electrode system was used in EIS measurement. The steel panel of the coating specimen was employed as the working electrode, while a platinum net and saturated calomel electrode were used as the counter electrode and reference electrode, respectively. A glass tube was clamped on the panel and 1.0% NaCl solution was added to immerse the electrodes. A Gamry Reference 600 Spectroscope (Warminster, PA, USA) was used to collect the EIS data and was presented as impedance vs. frequency curves. In addition, salt spray exposure (ASTM B117) was employed as an accelerated durability test to evaluate the long-term durability of the nanofiller-reinforced coatings.

#### 2.3.2. Abrasion Resistance

To examine the wear resistance of the developed coatings, a Taber abraser test was conducted in accordance with the ASTM D4060 standard. The abrasion resistance was characterized as the mass loss in a certain number of abrasive cycles under applied loads. In this study, each specimen was abraded by two CS-10 wheels with a rotational speed of 72 rpm for 1000 cycles while under a load of 1000 g during the test. During the test, the abrading wheels created a circular abrasion mark with an area of 30 cm^2^ on the coating as the surface layer was removed.

#### 2.3.3. Tensile Property

A coupon test was employed to examine the tensile properties of the developed nanocomposites. The tensile test was operated by a Shimadzu EZ-X tester (Columbia, MD, USA) by following the ASTM D638 standard. Tensile strength was applied to pull the specimen until the specimen broke in the narrow section. During the test, the loading rate was maintained at 1 mm/min, while real-time tensile strength and strain were recorded.

## 3. Results

### 3.1. Particles Shape and Size (TEM)

**Figure 3:**
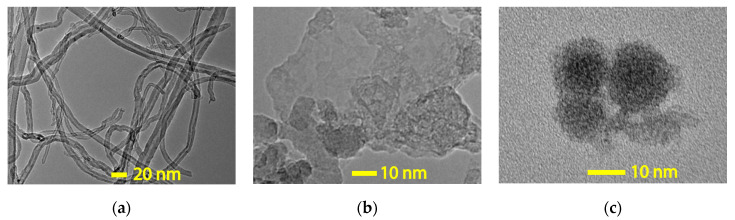
Transmission electron microscopy (TEM) photograph of (**a**) carbon nanotubes, (**b**) graphene nanoplatelets, and (**c**) fullerene-C60 particles.

Transmission electron microscopy (TEM) photographs of the as-received raw CNT, GNP, and C60 nanoparticles is shown in Figure 3a–c, respectively. The TEM graphs at high resolution allow one to observe the nanoparticles virtually. The observation was used to characterize the micro-structures of three different nanoparticles.

As shown in Figure 3 it is clear that the three types of nanoparticles can be defined as zero (C60), one (CNT), or two-dimensional (GNP) materials, which indicate that there are 0, 1, or 2 dimensions that are larger than 100 nm, respectively. This observation shows a strong agreement with the assumption that motivated the study, as part of the project objective was to investigate the reinforcing properties provided by the nanoparticles due to their unique shapes. A cylindrical shape was observed in the carbon nanotube (CNT) with a diameter around 15 to 20 nm. The graphene nanoplatelets (GNP) were stacked in single-layer sheets with an extremely small thickness. Furthermore, a homogeneous size distribution of the spherical particles was identified in fullerene-C60, with an average diameter around 20 nm.

### 3.2. Characterization of the Possible Chemical Reaction between Polymer Matrix and Nanofiller

**Figure 4:**
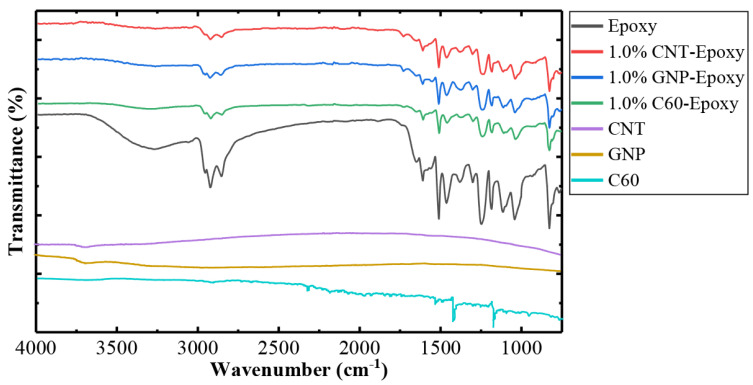
FTIR analysis of the nanocomposites with varied carbon nanofillers.

The nanofillers and nanocomposites were characterized using FTIR analysis. FTIR spectra of nanofillers and nanocomposites are illustrated in Figure 4, while the nanocomposites with 1.0% CNT, GNP, and C60 were selected to represent the filler/epoxy composites. The data were compared to neat epoxy to understand the possible chemical reaction between polymers and nanofillers.

To examine the potential of hydrogen bonding between the nanofiller and epoxy resin during the dispersion process, FTIR spectra were used to examine the existence of hydroxyl groups on each of the nanofillers’ surfaces. As presented in Figure 4, both raw CNTs and GNPs have no significant absorption peaks; however, the broad band at 3682 cm^−1^ indicates the presence of an O–H bond, which was contributed by an unbound or free hydroxyl group (moisture) attached to the nanofiller surfaces [27,28]. On the other hand, this bond was not observed in C60 particles; instead, several narrow peaks that represent C–C bonds were obtained at 1180 and 1427 cm^−1^ [29].

As illustrated in Figure 4, several peaks were identified to help characterize the neat epoxy and nanocomposites. The peak at 2920 cm^−1^ was due to the C–H stretch of the polymer backbone, and the peaks at 1607 and 1456 cm^−1^ were used to characterize the DGEBA epoxy resin that corresponds to the aromatic ring stretching of C=C. The disappearance of a peak around 913 cm^−1^ for all the tested samples indicates the ring opening polymerization during crosslinking, which reflects the curing process [30,31,32]. Close observation of the FTIR spectra, as illustrated in Figure 4, revealed that the characteristic peaks for all the samples were identical, as there were no changes in the peak value, suggesting that there was no new chemical bonding between the polymer and nanofillers [33].

### 3.3. Particle Size Distribution of Nanofillers and Interfacial Bonding to Polymer Matrix

**Figure 5:**
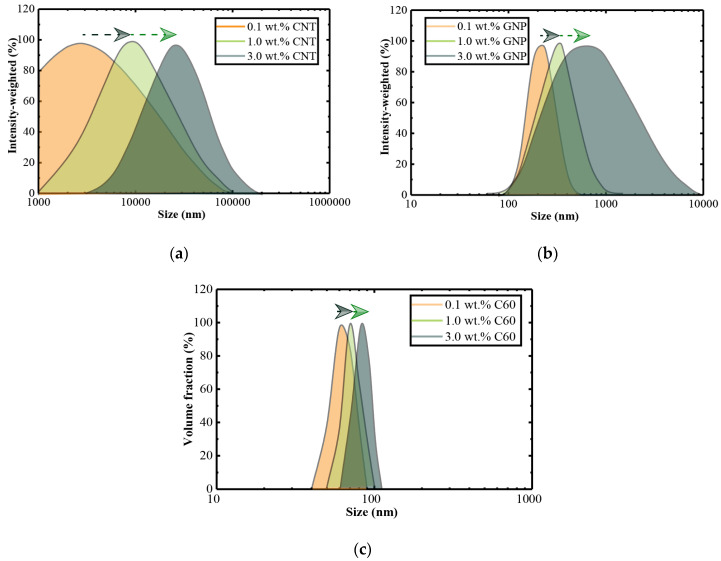
Particle size distribution of (**a**) carbon nanotubes (CNTs), (**b**) graphene nanoplatelets (GNPs), and (**c**) fullerene-C60 nanocomposites.

One of the most important factors in the nanoparticle’s reinforcement is the dispersion state of nanofillers. The size of nanoparticles can affect the reinforcement in the nanocomposites as larger aggregates create more severe defects, which leads to longer micro-cracks [3]. To examine the dispersibility of each nanofiller, the nano-particle size distributions were investigated by dynamic light scattering (DLS) with a submicron particle sizer (Nicomp 380, PSS Nicomp, Santa Barbara, CA, USA). Figure 5 shows the relation between the particle size distribution and weight concentration for the different nanofillers. As mentioned earlier, a similar dispersion procedure was used in each nanofiller, so the effect of the nanofiller’s shape on the tendency to format agglomerates could be observed.

The particle size distribution of the nanofillers mostly ranged from 10^3^ to 10^5^, 10^2^ to 10^4^, and 10^2^ to 10^3^ for CNT, GNP, and fullerene-C60, respectively (Figure 5). The results clearly indicated that, from 0.1 to 3.0 wt.%, the largest particle size was always observed in CNT groups; for example, the average size of 1.0 wt.% of CNT was around 2300 nm, while those of GNP and fullerene-C60 were 230 and 63 nm, respectively. The most significant increase in average size due to the increased weight content was also observed in CNT groups. These results indicated that CNT has the highest tendency to form agglomeration within the tested concentrations. On the other hand, for GNP particles, the changes in size distribution behaved differently. The average size increased due to the increased concentration of particles. However, large agglomerations were only observed from 1.0 to 3.0 wt.% groups, and no significant changes were observed from 0.1 to 1.0 wt.% groups. The results indicated that a severe agglomeration of GNP would take place when the weight content was larger than 1.0 wt.%. The results clearly indicated that fullerene-C60 has the least tendency to form agglomerations, as the average particle size did not significantly increase with the increase in concentration of the nanofiller. Additionally, compared to CNT and GNP, a much narrower size distribution was observed, in which the particles were 10^2^ to 10^3^ nm in size.

In conclusion, within the tested concentration, the 1D materials (CNT) have the highest tendency to form agglomeration, followed by the 2D materials (GNP), where the nanoplatelets stack up after 1.0 wt.%. The 0D particles (fullerene-C60) have the least tendency to form agglomeration, as no significant increases were observed in the particle size.

**Figure 6:**
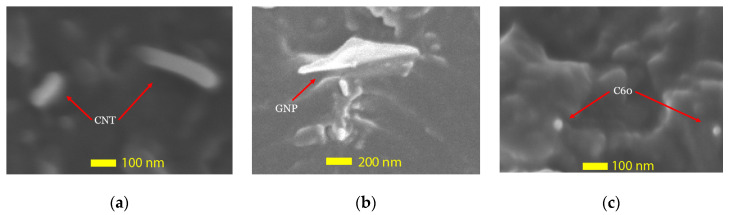
SEM images showing the interfacial bonding between nanoparticles and epoxy resin. (**a**) carbon nanotubes, (**b**) graphene nanoplatelets, and (**c**) fullerene-C60 particles.

Figure 6 illustrates the interfacial bonding between nanofillers and polymer matrices. The nanofillers were surrounded by the polymer, leading to effective reinforcement. Nanoparticles with strong bonding are shown as examples in Figure 6a–c. In Figure 6a, the CNT was embedded into epoxy at the end, which explains the improvement in the mechanical properties of the CNT/epoxy matrix. Similar interfacial interaction was observed in GNP and C60 groups; it could be easily observed that some nanofillers were wrapped with the epoxy resin and partially pulled out from the fracture surface.

### 3.4. Corrosion Barrier Performance of the Nanofiller-Reinforced Coatings

**Figure 7:**
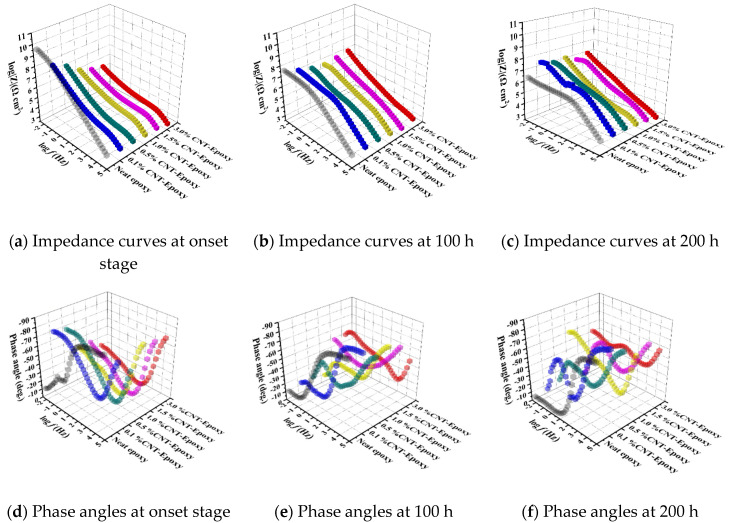
Bode plots of CNT/epoxy coatings: (**a**,**d**) Before, (**b**,**e**) 100, and (**c**,**f**) 200 h after exposure.

**Figure 8:**
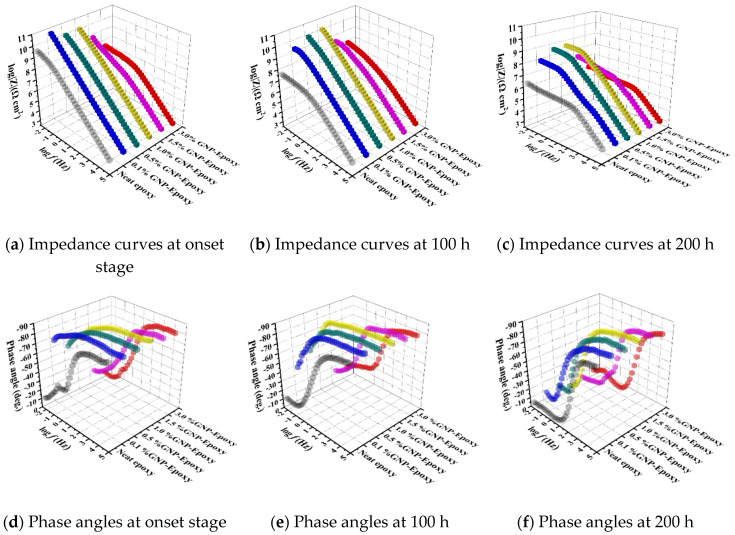
Bode plots of GNP/epoxy coatings: (**a**,**d**) Before, (**b**,**e**) 100, and (**c**,**f**) 200 h after exposure.

**Figure 9:**
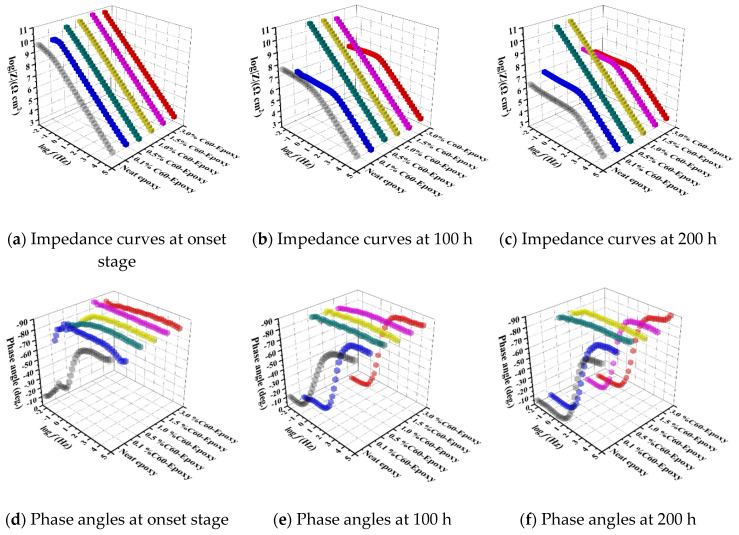
Bode plots of fullerene C60 (C60)/epoxy coatings: (**a**,**d**) Before, (**b**,**e**) 100, and (**c**,**f**) 200 h after exposure.

This section discusses the impacts of different carbon-based nanofillers as a reinforcement on the corrosion barrier performance of the nanocomposites in both the short term and long term, as summarized and shown in Figure 7, Figure 8 and Figure 9, where the combination of EIS and B117 salt fog tests were conducted for the short- and long-term performance.

#### 3.4.1. Short-term Corrosion Barrier Performance

(a) Neat epoxy as the references: At the onset of exposure, the obtained results suggested that the pure epoxy had good corrosion resistance. However, the bending in the low-frequency region of the Bode diagram indicated that the coating film could not act as an intact layer to protect the substrate. Researchers [27,34,35] suggested that neat epoxy could potentially generate micro-pores, which allowed the electrolyte to penetrate into the coating and contact with the metallic substrate. The reduced barrier performance eventually leads to a shortened service life.

(b) CNT/epoxy: Figure 7a–d show the impedance and phase angle plots for the coatings reinforced with CNT. In the lower-frequency region, a decrease in the impedance value was obtained, and the impedance value gradually degraded as the CNT content in the composite increased. Apparently, the highly conductive network formed by CNT reduced the electrical resistance of the coatings, while the thickness of the polymer interface layer for electron hopping also decreased [11,36]. The breakpoint frequency at −45° shifted to the high-frequency region in the phase angle diagram, suggesting that the capacitance of the coating layer was significantly reduced compared to the pure epoxy group.

(c) GNP/epoxy: As illustrated in Figure 8a–d, by adding a low content of GNP, the corrosion resistance of epoxy coatings was significantly improved. Compared to pure epoxy samples, nanocomposites containing 0.1, 0.5, and 1 wt.% GNPs showed a higher impedance modulus. The resistance of corrosion for the 0.1% GNP-epoxy group was higher than those of the groups with 0.5% and 1.0% of GNPs. Material degradation in corrosion resistance was observed in samples with higher concentrations of graphene, and the Z_mod_ value began to decrease in composites with 1.5% by weight of GNP. Unlike pure epoxy and CNT/epoxy, 0.1%, 0.5%, and 1.0% GNP-epoxies had a slope of −1 in the impedance diagram, while the values reached 90° in the phase angle plot. This result suggests that the GNPs worked as barriers to the corrosive media; moreover, the existence of well-distributed graphene nanosheets in these groups effectively improved the barrier properties of the polymeric films. In this case, these GNP/epoxy coatings offered excellent corrosion protection to the substrate, and this observation was consistent with the other researchers’ studies [37,38]. However, with a higher amount of GNPs, 1.5% and 3.0%GNP-epoxy groups behaved differently. The specimens showed a degraded corrosion resistance instead of improving the performance of the composites. As presented in Figure 8a–d, a lower phase angle was obtained at 0.01 Hz in the 1.5%GNP-epoxy and 3.0%GNP-epoxy groups, and the values were smaller than those of the groups with less GNPs or even the neat epoxy; the result suggests that the high-GNP-content groups had weaker anti-corrosion properties as compared to the others. This phenomenon could be caused by the aggregation of nanoparticles when an excessive amount of GNPs was added in the polymer matrix.

(d) C60/epoxy: Figure 9a,d present Bode plots for the steel substrate coated with fullerene-C60/epoxy composite coatings. Evidently, with a low content of C60 particles, the 0.1%C60-epoxy exhibited no significant enhancement in anti-corrosion performance, as compared to the neat epoxy. Besides, it turned out that a higher amount of C60 nanoparticles strengthened the coating, as the 0.5% to 3.0%C60-epoxy groups exhibited straight lines with high |Z|_0.01Hz_ values in the impedance plot, suggesting the coating films had excellent barrier performance and provided perfect resistance to corrosion.

#### 3.4.2. Long-Term Corrosion Barrier Performance

(a) Neat epoxy after 100/200 h accelerated durability tests: The long-term performance of the neat epoxy coating after 100/200 h exposure of salt spray, illustrated by the Bode plots Figure 7, Figure 8 and Figure 9, showed a good agreement with the previous prediction of corrosion protection performance. The neat epoxy coating displayed a severe degradation in corrosion protection property. The impedance value at 0.01 Hz (|Z|_0.01Hz_) dropped about two and a half orders, indicating that the barrier properties of the coating had been decreased. Thus, the minimum region of the phase angle curve approached the high-frequency region, suggesting that the neat epoxy coating degraded during the exposure, which led to reduced corrosion protection properties, as also confirmed by other researchers [23].

(b) CNT/epoxy after 100/200 h accelerated durability tests: Figure 7b–f plots the CNT/epoxy coatings after accelerated durability tests using the salt spray. As mentioned in the previous section, a decrease in impedance value at the low-frequency region represents a reduction in barrier properties of the coatings, which leads to a weaker corrosion resistance [21]. However, as confirmed elsewhere, this suggestion was not suitable for the coatings that contain CNT, in which a highly conductive network was built within the polymer matrix, and it led to a reduction in overall electrical resistance of the coating. The presence of carbon nanotubes not only acts as a barrier to the penetration of corrosive media, but also reduces the water absorption of the coating. Moreover, CNTs can increase the bonding strength between coating and substrate, which leads to an overall improvement in corrosion resistance [10,11]. The performance of CNT/epoxy coatings has confirmed the suggestion above. For 1.0%, 1.5%, and 3.0%CNT-epoxy groups, the impedance values at the lowest frequency in the Bode plot increased after exposure. As confirmed from the literature study [39], this phenomenon was attributed to the corrosion product layer formed by corrosion reactions. As the exposure time elapsed, the accumulated corrosion products formed a thin film on the substrate surface; hence, the radius of the capacitive impedance arc increased.

(c) GNP/epoxy after 100/200 h accelerated durability tests: As clearly illustrated in Figure 8b–f, the long-term exposure further emphasized that the addition of GNP nanosheets in epoxy resin could dramatically improve the corrosion protection and increase the durability. Among all the GNP/epoxy nanocomposites, the 0.1%, 0.5%, and 1.0%GNP-epoxy groups exhibited the best resistance to corrosion in terms of significant improvements in durability. With a slight decrease in the impendence modulus at 0.01 Hz, this suggests that the coating films with 0.1, 0.5, and 1.0 wt.% GNPs still offered an effective protecting layer for the substrates after the 100 h exposure. However, the phase angle at low frequency reduced, presenting the penetration of electrode media into the coating film. Similar to the performance at the fresh stage, the groups with a higher loaded graphene had little degraded corrosion resistance mostly contributed by the GNP agglomeration. Figure 8 shows that the groups, 1.5% and 3.0%GNP-epoxy, offered a fair long-term protection performance, which was identical to the neat epoxy.

(d) C60/epoxy after 100/200 h accelerated durability tests: As shown in Figure 9b–f, similar to the initial performance, the 0.1%C60-epoxy after 100 h of exposure behaved with the weakest resistance to corrosion, which further emphasized that the low-content C60 particles were not able to provide a noticeable reinforcement in barrier performance to epoxy resin. Furthermore, the |Z|_0.01Hz_ in the 3.0%C60-epoxy group was reduced, suggesting that the coating was damaged and delaminated during the salt fog exposure. This observation pointed out that a higher loading of C60 nanoparticles could offer a short-term improvement in anti-corrosion properties; however, they would not be able to provide enough durability against long-term exposure. When the exposure time increased up to 200 h, it is clear that 1.0%C60-epoxy and 1.5%C60-epoxy groups were damaged as the |Z|_0.01Hz_ values were significantly decreased. Meanwhile, the breakpoint of the phase angle shifted from low frequencies toward intermediate frequencies. On the contrary, the 0.5% and 1.0%C60-epoxy samples exhibited outstanding corrosion protection properties, despite the exposure duration. The impedance values of 0.5% and 1.0%C60-epoxy samples were maintained over 10^10^ Ω/cm^2^ after salt, while a straight line was observed in the impedance plot. Thus, these observations confirmed that the coating behaved as intact films to protect the substrate during the exposure, suggesting that the 0.5 and 1.0 wt.% of C60 nanoparticles significantly enhanced the anti-corrosion performance of the epoxy coating, with improved durability as well.

### 3.5. Abrasion Resistance of the Nanofiller-Reinforced Coatings

**Figure 10:**
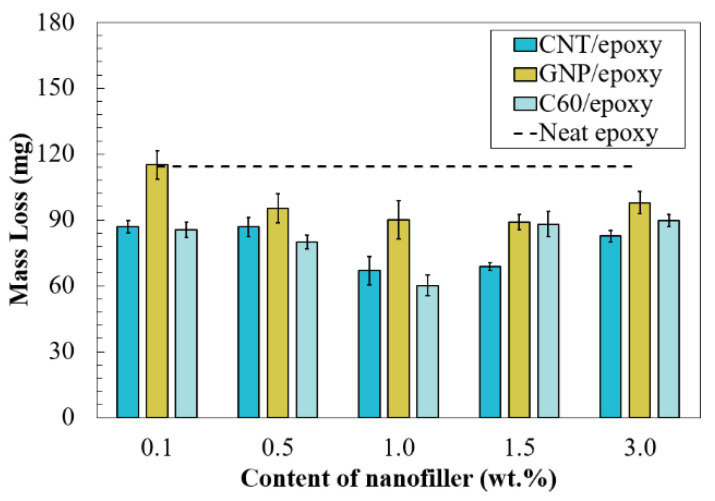
Mass loss of the nanocomposites with varied types of carbon nanofillers.

To evaluate the wear resistance of the prepared carbon-based nanofiller-reinforced epoxy coatings, the abrasion resistance test was performed, and wear mass loss was measured during the test. To clarify, less mass loss after the test indicates that the coating has stronger wear resistance. As displayed in Figure 10, the mass loss of the neat epoxy was 114 mg; in addition, compared to the neat epoxy, enhanced wear resistance was observed in the nanocomposites reinforced by CNT, GNP, or C60 particles. The generalizability of the research work suggested that the maximum reinforcement of abrasion resistance required a proper amount of nanofiller, leading to an improvement in the cohesion or strength of the coatings, and the ability to transfer the applied load during the abrasion process [40,41].

In general, the CNT/epoxy groups exhibited a remarkable improvement in abrasion resistance in all the tested concentrations, as reduced mass loss was observed in all the tested concentrations. It is apparent that the CNT/epoxy system showed a maximum enhancement with a content of 1.0 wt.%, as the mass loss was reduced by around 40%. The values of the CNT/epoxy composite with 1.5 and 1.0 wt.% were very close, indicating that the 1.0 to 1.5 wt.% content of CNT provided strong reinforcement in the epoxy matrix. However, material degradation was observed at samples with a higher concentration of CNT. One reasonable suggestion is that the degradation is caused by the severe agglomeration of CNT, which was confirmed in the particle size distribution test.

An improvement in abrasion resistance was obtained in all prepared C60-reinforced nanocomposites. The maximum improvement in abrasion resistance was obtained in 1.0%C60-epoxy, and the weight of mass loss was 60 mg, which was reduced by 47%. For the composites containing other contents of fullerene-C60, the mass loss was generally higher than that of the coating with 1.0 wt.% of fullerene-C60. However, improved abrasion resistance was observed by comparing to neat epoxy; the mass loss of the other nanocomposites ranged from 80 to 90 mg. Generally, regardless of the types of nanofillers, degradation of wear resistance performance was obtained in the nanocomposites containing a high amount of nanofillers.

Compared to other nanofillers, a higher mass loss was observed in the GNP/epoxy coatings, regardless of the concentrations of nanoparticles. Close to the CNT/epoxy and C60/epoxy, a similar tendency was observed in GNP/epoxy, where the lowest mass loss was observed in 1.0 and 1.5 wt.% groups, and yet, the mass loss increased with the higher-content GNP, revealing that material degradation was obtained.

**Figure 11:**
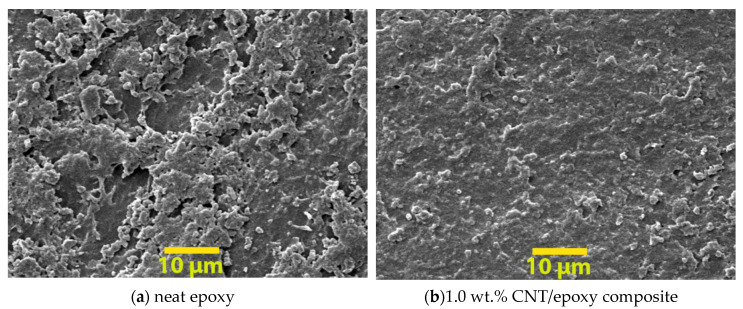

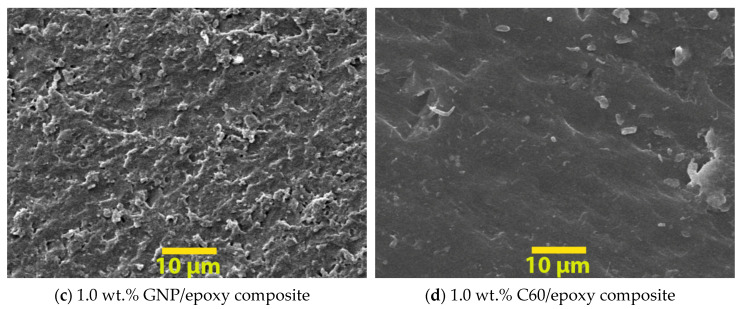
SEM images of abraded surfaces for typical samples: (**a**–**d**).

SEM images of the abraded surface were utilized to identify the surface properties of the coatings after the Taber abraser test; thus, the neat epoxy and nanocomposite with 1.0 wt.% of nanofillers were used as examples in Figure 11. Apparently, the neat epoxy (see Figure 11a) has a rougher surface, as a massive proportion of cracks and cleavages was observed, as compared to other cases (see Figure 11b–d), indicating a plastic deformation with low wear resistance of the neat epoxy coating. On the other hand, smoother surfaces were observed in nanofiller/epoxy groups, with the reduction in both size and number of cracks and grooves, suggesting that the nanofillers strengthened the nanocomposites when under abrasive loads.

### 3.6. Tensile Behavior of the Nanofiller-Reinforced Coatings

Studying tensile properties could be used to evaluate the damage tolerance of the nanofiller-reinforced epoxy. The coupon tensile test was utilized to examine the tensile properties of the nanofiller/epoxy composites. The analysis was performed by discussing the tensile strength, maximum strain, and Young’s modulus of specimens, by following ASTM D638.

**Figure 12:**
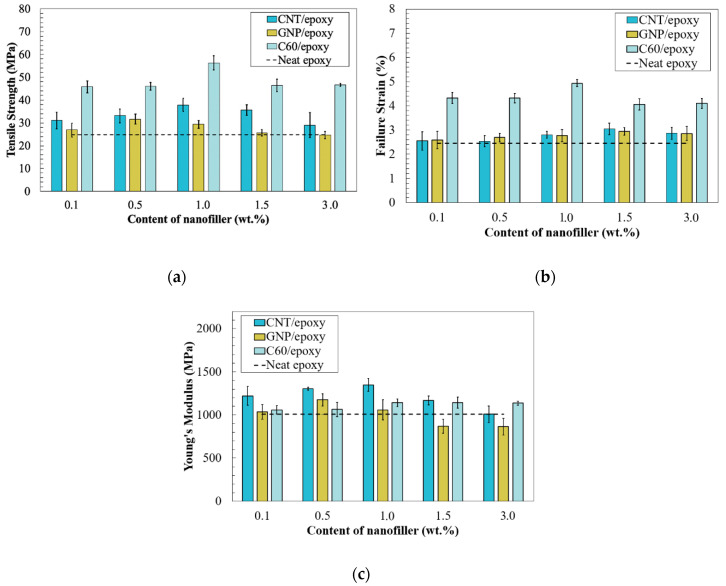
(**a**) Tensile strength, (**b**) failure strain, and (**c**) Young’s modulus of nanofiller/epoxy composites.

Figure 12a shows the tensile strength of nanofiller/epoxy coatings with different weight contents. The neat epoxy was used as the reference and the tensile strength was around 24 MPa. For the CNT/epoxy groups, the strength was gradually increased and reached the maximum stress of 38 MPa at 1.0 wt.% of CNT, which increased 55% by comparing to the pure epoxy group (24 MPa). In the GNP/epoxy groups, the highest stress value was obtained in the 0.1 wt.% group, in which a 40% improvement was observed. Moreover, the results suggested that the C60/epoxy had the maximum improvement in tensile strength, as the tensile strength was above 45 MPa in all the C60-reinforced coatings, regardless of the weight content of nanofillers. Identical to the CNT/epoxy groups, the tensile strength was gradually increased and reached 56 MPa in the 1.0% C60-epoxy, which increased 130% compared to the neat epoxy composite. In all the tested nanocomposites, the results also suggested that degradation occurred in the nanocomposite with heavily loaded nanofillers; thus, the strength started to decrease when a higher content of nanoparticles was added into the matrix, and the lowest strength was obtained in composites with 3.0 wt.% of nanoparticles, regardless of the type of nanofillers.

The failure strain of the nanocomposite coating is presented in Figure 12b. Identical to the tensile strength, the presence of C60 nanoparticles led to a significantly increased strain, and improvement was observed in all C60/epoxy samples. The failure strain of the neat epoxy was 2.4%, while those of all the C60/epoxy groups were higher than 4.0%. In addition, the highest strain value was observed in the sample with 1.0 wt.% of C60 particles, which was one-time higher than that of the neat epoxy. In addition, the failure strains started to decrease when the concentration of fullerene-C60 particles was higher than 1.0 wt.%. On the other hand, no significant reinforcement was observed in the samples containing CNTs or GNPs; however, it is worth mentioning that a gradual increase in the failure strain was observed in the CNT and GNP groups and the maximum value was obtained in the 3.0 wt.% groups.

However, the results of Young’s modulus were different from the max tensile strength and strain (see Figure 12c). The highest reinforcement was obtained in CNT/epoxy groups, the maximum Young’s modulus was observed in the 1.0%CNT-epoxy groups, and the values started to decrease when the CNT content exceeded 1.0 wt.%. The 1%CNT-epoxy group had a Young’s modulus value of 1350 MPa, which increased by 34% compared to pure epoxy. By contrast, only a slight reinforcement was observed with the addition of C60/epoxy. The value of Young’s modulus was gradually increased from 0.1 to 1.0%C60-epoxy groups, and the value was maintained when an even higher content of C60 was added (1.5 and 3.0 wt.%). On the other hand, with the same weight content of nanofillers, the lowest Young’s modulus values were generally found in GNP/epoxy groups, while Young’s modulus of 1.5 and 3.0 wt.% GNP-epoxy was even lower than that of the neat epoxy group.

**Figure 13:**
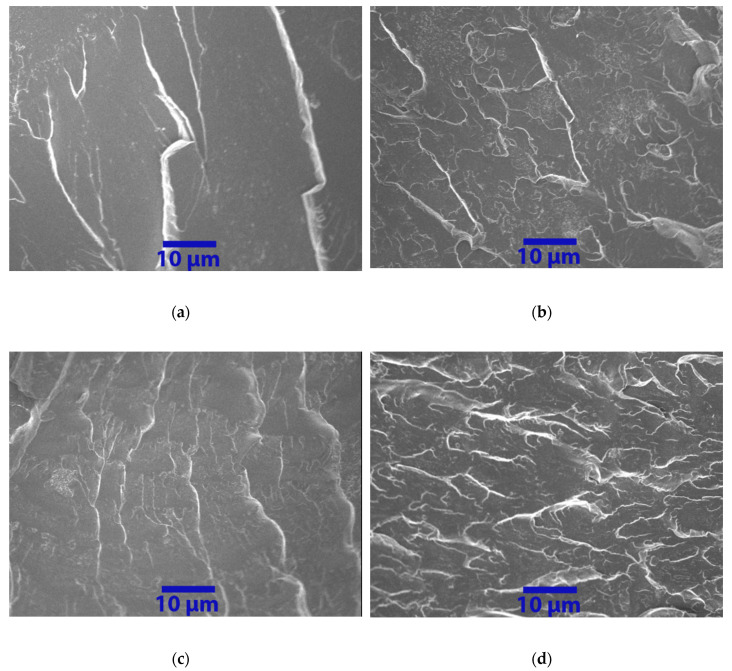
Fracture surface of (**a**) neat epoxy, (**b**) CNT/epoxy composite containing 1.0 wt.% of carbon nanotubes, (**c**) GNP/epoxy composite containing 1.0 wt.% of graphene nanoplatelets, and (**d**) C60/epoxy composite containing 1.0 wt.% of C60.

The morphologies of fracture surfaces for specimens under tension are illustrated in Figure 13a–d. The SEM image of neat epoxy presented a typical brittle fracture surface, as a smooth surface with large cracks was observed, suggesting the neat epoxy has a weak resistance to impact and low-fracture toughness. By contrast, an improved fracture surface was observed in all the tested nanofiller/epoxy composites, especially for the composites containing C60 (see Figure 13d), which exhibited a much rougher fracture surface containing very compacted cleavages, suggesting an increased energy absorption and improved fracture toughness. The results showed a strong agreement with the observation from the tensile test, as the C60/epoxy has the highest failure strain. Similar observations were found in the CNT and GNP groups, which can be a good indication of the strong adhesion between the reins and well-dispersed nanofillers in the polymer matrix.

## 4. Conclusions

Epoxy coatings with differently shaped carbon nanofillers (CNT, GNP, and C60) were successfully fabricated and characterized. The tribological, mechanical, and electrical properties were studied to investigate its potential as a high-performance coating for protecting metal structures. Nano-reinforced epoxy coating systems were fabricated with various amounts of nanofillers (0.1, 0.5, 1.0, 1.5, and 3.0 wt.%). Based on the experimental results, the following main findings were summarized:

- By comparing the EIS data before and after exposure, the highest corrosion resistance was obtained in the fullerene-C60-reinforced epoxy coatings. Strong corrosion resistance was also observed in the GNP/epoxy coating at the fresh stage, but the Z_mod_ value started to decrease after 100 h of exposure. The lowest reinforcement effect was found in the CNT/epoxy coating in both short-term and long-term cases.
- Strong reinforcement of abrasion resistance was obtained with the presence of C60 and CNT nanoparticles, and the mass loss of these two composites was very close. The addition of GNP particles also increased the abrasion resistance, but their reinforcement effects were weaker than those of CNT and fullerene-C60.
- A significant improvement in adhesion between the coating and substrate was only observed in the CNT/epoxy group.
- The highest tensile strength was obtained in the C60/epoxy composite, while the GNP/epoxy had the lowest reinforcement, and the CNT/epoxy was in between these two coatings. A substantial improvement in failure strain was only observed in the fullerene-C60/epoxy group, and the highest Young’s modulus was observed in the CNT/epoxy group.
- The results of this study suggested that the geometric shape of nanofillers plays a vital role in nano-reinforcement, as the linearly shaped CNT results in a strong improvement in mechanical properties, while the 2D nanosheet (GNP) significantly increased the barrier properties. In addition, the spherically shaped nanofiller (fullerene) has the least potential to form agglomeration, which leads to an overall enhancement in nanocomposites. Meanwhile, the addition of 0.5–1.5 wt.% nanofillers will potentially result in the highest improvement in both electrical behaviors and mechanical properties.
